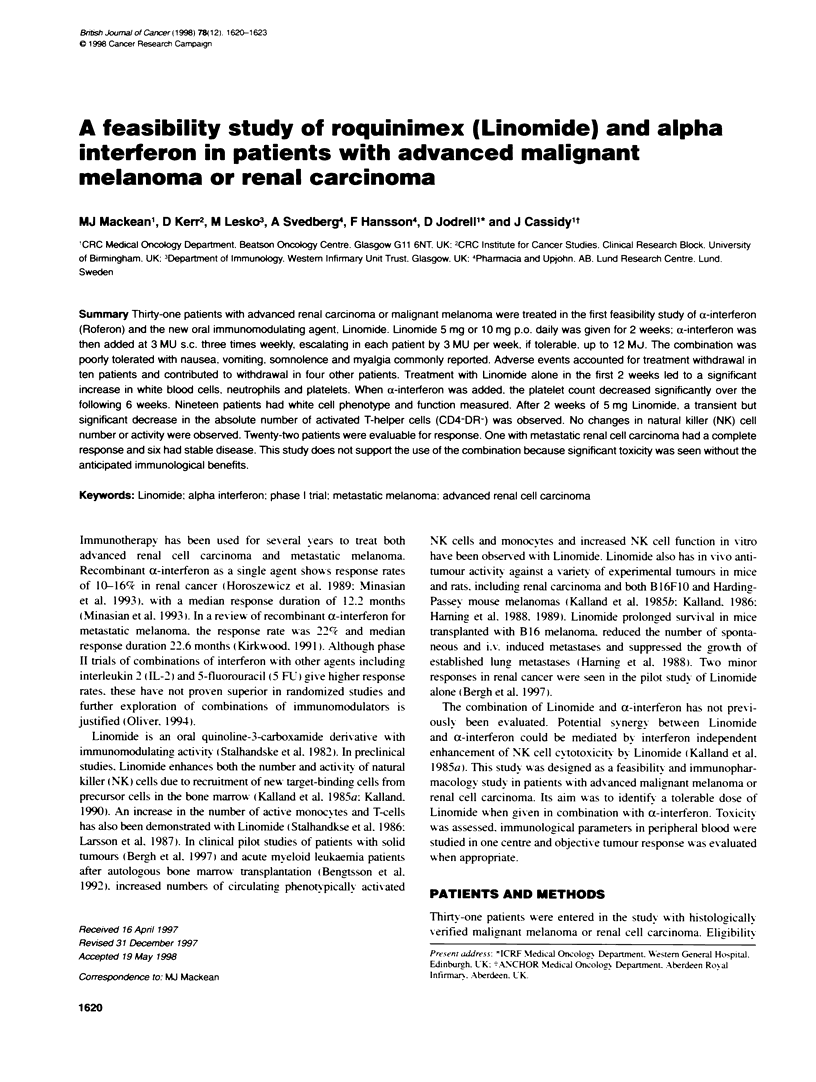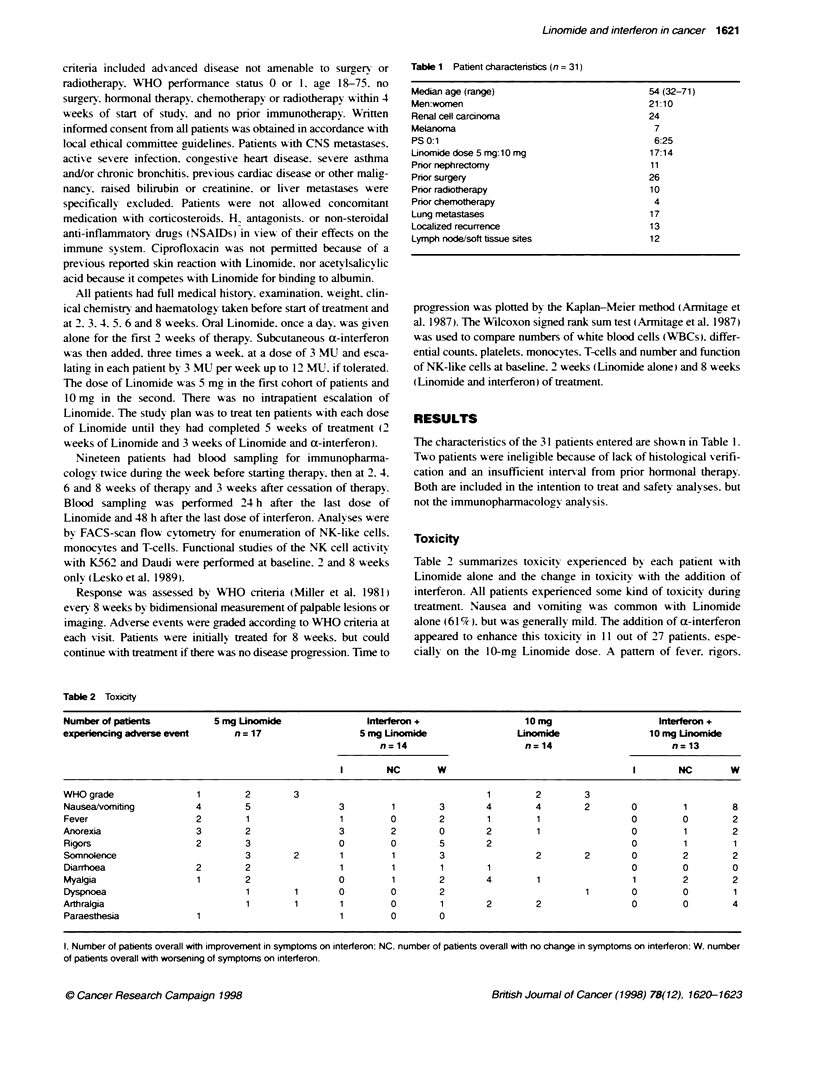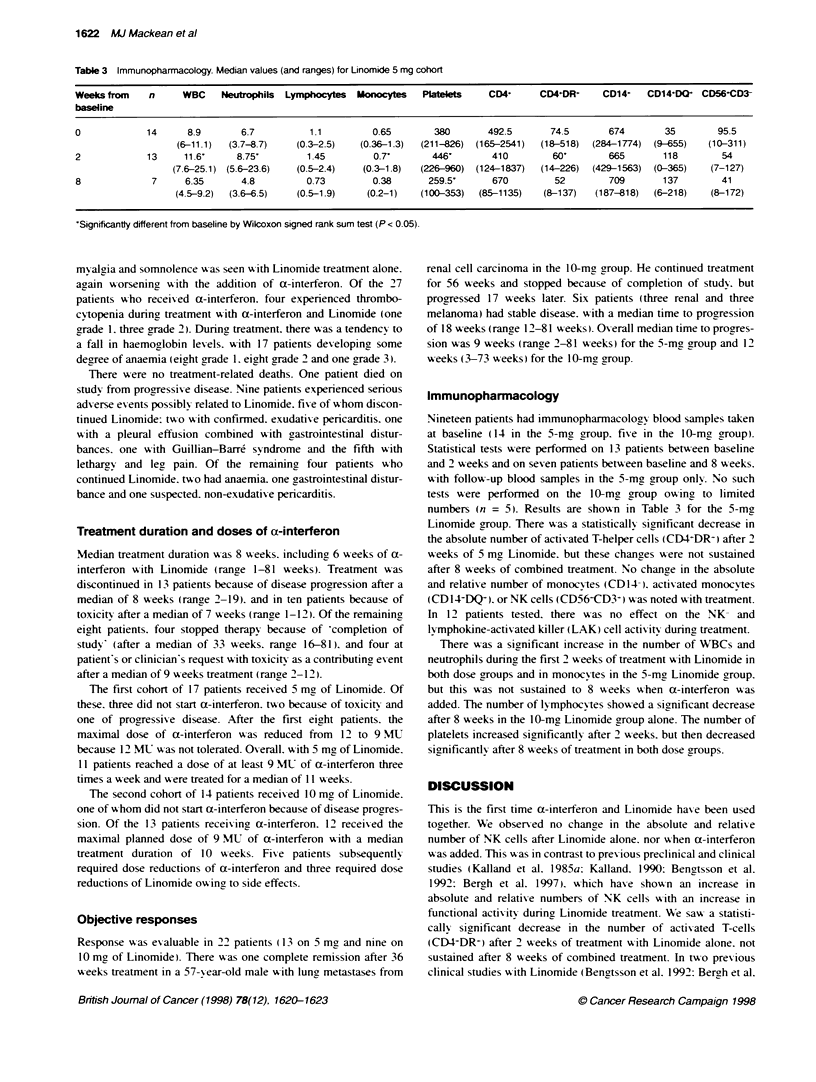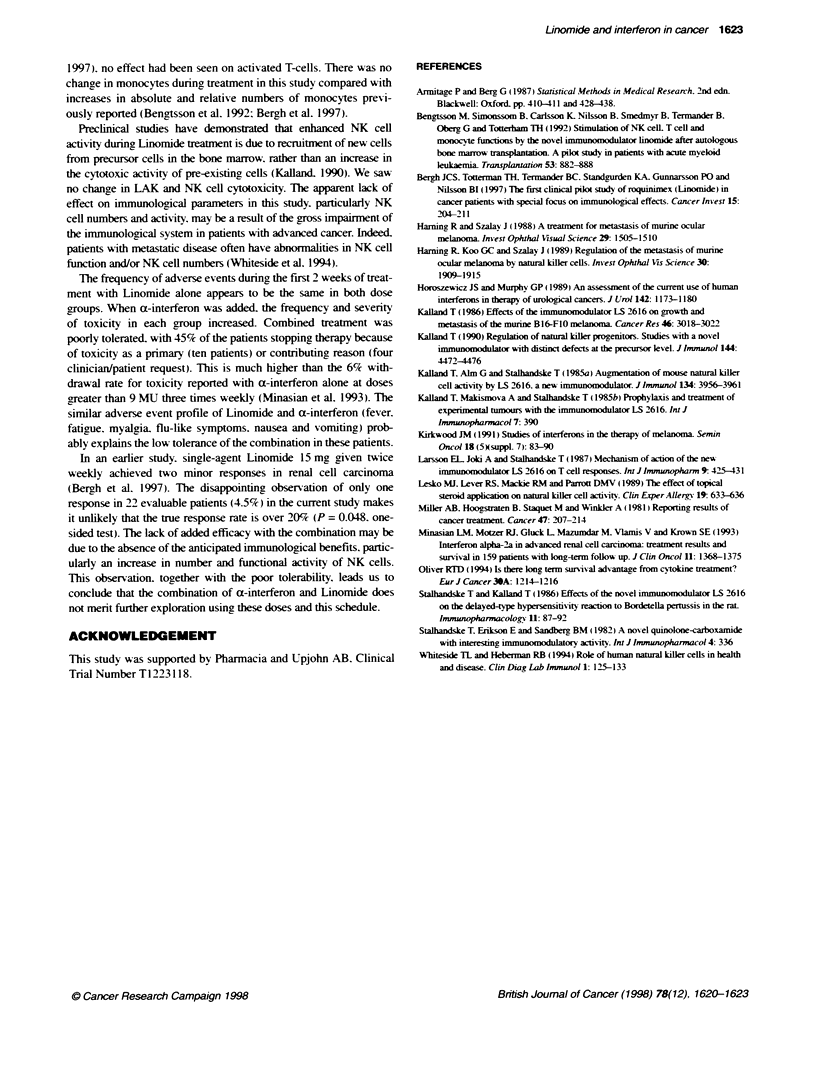# A feasibility study of roquinimex (Linomide) and alpha interferon in patients with advanced malignant melanoma or renal carcinoma.

**DOI:** 10.1038/bjc.1998.732

**Published:** 1998-12

**Authors:** M. J. Mackean, D. Kerr, M. Lesko, A. Svedberg, F. Hansson, D. Jodrell, J. Cassidy

**Affiliations:** CRC Medical Oncology Department, Beatson Oncology Centre, Glasgow, UK.

## Abstract

Thirty-one patients with advanced renal carcinoma or malignant melanoma were treated in the first feasibility study of alpha-interferon (Roferon) and the new oral immunomodulating agent, Linomide. Linomide 5 mg or 10 mg p.o. daily was given for 2 weeks; alpha-interferon was then added at 3 MU s.c. three times weekly, escalating in each patient by 3 MU per week, if tolerable, up to 12 MJ. The combination was poorly tolerated with nausea, vomiting, somnolence and myalgia commonly reported. Adverse events accounted for treatment withdrawal in ten patients and contributed to withdrawal in four other patients. Treatment with Linomide alone in the first 2 weeks led to a significant increase in white blood cells, neutrophils and platelets. When alpha-interferon was added, the platelet count decreased significantly over the following 6 weeks. Nineteen patients had white cell phenotype and function measured. After 2 weeks of 5 mg Linomide, a transient but significant decrease in the absolute number of activated T-helper cells (CD4+DR+) was observed. No changes in natural killer (NK) cell number or activity were observed. Twenty-two patients were evaluable for response. One with metastatic renal cell carcinoma had a complete response and six had stable disease. This study does not support the use of the combination because significant toxicity was seen without the anticipated immunological benefits.


British Journal of Cancer (1998) 78(12). 1620–1623

© 1998 Cancer Research Campaign

### A feasibility study of roquinimex (Linomide) and alpha interferon in patients with advanced malignant melanoma or renal carcinoma

**MJ Mackean[1], D Kerr[2], M Lesko[3], A Svedberg[4], F Hansson[4], D Jodrell[1*] and J Cassidy[1†]**

[1]CRC Medical Oncology Department. Beatson Oncology Centre. Glasgow G11 6NT. UK: [2]CRC Institute for Cancer Studies. Clinical Research Block. University of Birmingham. UK: [3]Department of Immunology. Western Infirmary Unit Trust. Glasgow. UK: [4]Pharmacia and Upjohn. AB. Lund Research Centre. Lund. Sweden

**Summary** Thirty-one patients with advanced renal carcinoma or malignant melanoma were treated in the first feasibility study of α-interferon (Roferon) and the new oral immunomodulating agent, Linomide. Linomide 5 mg or 10 mg p.o. daily was given for 2 weeks; α-interferon was then added at 3 MU s.c. three times weekly, escalating in each patient by 3 MU per week, if tolerable, up to 12 MU. The combination was poorly tolerated with nausea, vomiting, somnolence and myalgia commonly reported. Adverse events accounted for treatment withdrawal in ten patients and contributed to withdrawal in four other patients. Treatment with Linomide alone in the first 2 weeks led to a significant increase in white blood cells, neutrophils and platelets. When α-interferon was added, the platelet count decreased significantly over the following 6 weeks. Nineteen patients had white cell phenotype and function measured. After 2 weeks of 5 mg Linomide, a transient but significant decrease in the absolute number of activated T-helper cells (CD4+DR+) was observed. No changes in natural killer (NK) cell number or activity were observed. Twenty-two patients were evaluable for response. One with metastatic renal cell carcinoma had a complete response and six had stable disease. This study does not support the use of the combination because significant toxicity was seen without the anticipated immunological benefits.

**Keywords:** Linomide; alpha interferon; phase I trial; metastatic melanoma; advanced renal cell carcinoma

Immunotherapy has been used for several years to treat both advanced renal cell carcinoma and metastatic melanoma. Recombinant α-interferon as a single agent shows response rates of 10–16% in renal cancer (Horoszewicz et al. 1989; Minasian et al. 1993), with a median response duration of 12.2 months (Minasian et al. 1993). In a review of recombinant α-interferon for metastatic melanoma, the response rate was 22% and median response duration 22.6 months (Kirkwood, 1991). Although phase II trials of combinations of interferon with other agents including interleukin 2 (IL-2) and 5-fluorouracil (5 FU) give higher response rates, these have not proven superior in randomized studies and further exploration of combinations of immunomodulators is justified (Oliver, 1994).

Linomide is an oral quinoline-3-carboxamide derivative with immunomodulating activity (Stalhandske et al. 1982). In preclinical studies, Linomide enhances both the number and activity of natural killer (NK) cells due to recruitment of new target-binding cells from precursor cells in the bone marrow (Kalland et al. 1985*a*; Kalland, 1990). An increase in the number of active monocytes and T-cells has also been demonstrated with Linomide (Stalhandkse et al. 1986; Larsson et al. 1987). In clinical pilot studies of patients with solid tumours (Bergh et al. 1997) and acute myeloid leukaemia patients after autologous bone marrow transplantation (Bengtsson et al. 1992), increased numbers of circulating phenotypically activated NK cells and monocytes and increased NK cell function in vitro have been observed with Linomide. Linomide also has in vivo antitumour activity against a variety of experimental tumours in mice and rats, including renal carcinoma and both B16F10 and Harding-Passey mouse melanomas (Kalland et al. 1985*b*; Kalland, 1986; Harning et al. 1988, 1989). Linomide prolonged survival in mice transplanted with B16 melanoma, reduced the number of spontaneous and i.v. induced metastases and suppressed the growth of established lung metastases (Harning et al. 1988). Two minor responses in renal cancer were seen in the pilot study of Linomide alone (Bergh et al. 1997).

The combination of Linomide and α-interferon has not previously been evaluated. Potential synergy between Linomide and α-interferon could be mediated by interferon independent enhancement of NK cell cytotoxicity by Linomide (Kalland et al. 1985*a*). This study was designed as a feasibility and immunopharmacology study in patients with advanced malignant melanoma or renal cell carcinoma. Its aim was to identify a tolerable dose of Linomide when given in combination with α-interferon. Toxicity was assessed, immunological parameters in peripheral blood were studied in one centre and objective tumour response was evaluated when appropriate.

### PATIENTS AND METHODS

Thirty-one patients were entered in the study with histologically verified malignant melanoma or renal cell carcinoma. Eligibility

*Received 16 April 1997*
*Revised 31 December 1997*
*Accepted 19 May 1998*

*Correspondence to:* MJ Mackean

*Present address*: *ICRF Medical Oncology Department. Western General Hospital. Edinburgh. UK: †ANCHOR Medical Oncology Department. Aberdeen Royal Infirmary. Aberdeen. UK.

criteria included advanced disease not amenable to surgery or radiotherapy. WHO performance status 0 or 1, age 18–75, no surgery, hormonal therapy, chemotherapy or radiotherapy within 4 weeks of start of study, and no prior immunotherapy. Written informed consent from all patients was obtained in accordance with local ethical committee guidelines. Patients with CNS metastases, active severe infection, congestive heart disease, severe asthma and/or chronic bronchitis, previous cardiac disease or other malignancy, raised bilirubin or creatinine, or liver metastases were specifically excluded. Patients were not allowed concomitant medication with corticosteroids, $H_2$ antagonists, or non-steroidal anti-inflammatory drugs (NSAIDs) in view of their effects on the immune system. Ciprofloxacin was not permitted because of a previous reported skin reaction with Linomide, nor acetylsalicylic acid because it competes with Linomide for binding to albumin.

All patients had full medical history, examination, weight, clinical chemistry and haematology taken before start of treatment and at 2, 3, 4, 5, 6 and 8 weeks. Oral Linomide, once a day, was given alone for the first 2 weeks of therapy. Subcutaneous α-interferon was then added, three times a week, at a dose of 3 MU and escalating in each patient by 3 MU per week up to 12 MU, if tolerated. The dose of Linomide was 5 mg in the first cohort of patients and 10 mg in the second. There was no intrapatient escalation of Linomide. The study plan was to treat ten patients with each dose of Linomide until they had completed 5 weeks of treatment (2 weeks of Linomide and 3 weeks of Linomide and α-interferon).

Nineteen patients had blood sampling for immunopharmacology twice during the week before starting therapy, then at 2, 4, 6 and 8 weeks of therapy and 3 weeks after cessation of therapy. Blood sampling was performed 24 h after the last dose of Linomide and 48 h after the last dose of interferon. Analyses were by FACS-scan flow cytometry for enumeration of NK-like cells, monocytes and T-cells. Functional studies of the NK cell activity with K562 and Daudi were performed at baseline, 2 and 8 weeks only (Lesko et al, 1989).

Response was assessed by WHO criteria (Miller et al, 1981) every 8 weeks by bidimensional measurement of palpable lesions or imaging. Adverse events were graded according to WHO criteria at each visit. Patients were initially treated for 8 weeks, but could continue with treatment if there was no disease progression. Time to progression was plotted by the Kaplan–Meier method (Armitage et al, 1987). The Wilcoxon signed rank sum test (Armitage et al, 1987) was used to compare numbers of white blood cells (WBCs), differential counts, platelets, monocytes, T-cells and number and function of NK-like cells at baseline, 2 weeks (Linomide alone) and 8 weeks (Linomide and interferon) of treatment.

**Table 1** Patient characteristics ($n$ = 31)

| | |
|---|---|
| Median age (range) | 54 (32–71) |
| Men:women | 21:10 |
| Renal cell carcinoma | 24 |
| Melanoma | 7 |
| PS 0:1 | 6:25 |
| Linomide dose 5 mg:10 mg | 17:14 |
| Prior nephrectomy | 11 |
| Prior surgery | 26 |
| Prior radiotherapy | 10 |
| Prior chemotherapy | 4 |
| Lung metastases | 17 |
| Localized recurrence | 13 |
| Lymph node/soft tissue sites | 12 |

### RESULTS

The characteristics of the 31 patients entered are shown in Table 1. Two patients were ineligible because of lack of histological verification and an insufficient interval from prior hormonal therapy. Both are included in the intention to treat and safety analyses, but not the immunopharmacology analysis.

### Toxicity

Table 2 summarizes toxicity experienced by each patient with Linomide alone and the change in toxicity with the addition of interferon. All patients experienced some kind of toxicity during treatment. Nausea and vomiting was common with Linomide alone (61%), but was generally mild. The addition of α-interferon appeared to enhance this toxicity in 11 out of 27 patients, especially on the 10-mg Linomide dose. A pattern of fever, rigors,

**Table 2** Toxicity

| Number of patients experiencing adverse event | 5 mg Linomide $n$ = 17 | | | Interferon + 5 mg Linomide $n$ = 14 | | | 10 mg Linomide $n$ = 14 | | | Interferon + 10 mg Linomide $n$ = 13 | | |
|---|---|---|---|---|---|---|---|---|---|---|---|---|
| | | | | I | NC | W | | | | I | NC | W |
| WHO grade | 1 | 2 | 3 | | | | 1 | 2 | 3 | | | |
| Nausea/vomiting | 4 | 5 | | 3 | 1 | 3 | 4 | 4 | 2 | 0 | 1 | 8 |
| Fever | 2 | 1 | | 1 | 0 | 2 | 1 | 1 | | 0 | 0 | 2 |
| Anorexia | 3 | 2 | | 3 | 2 | 0 | 2 | 1 | | 0 | 1 | 2 |
| Rigors | 2 | 3 | | 0 | 0 | 5 | 2 | | | 0 | 1 | 1 |
| Somnolence | | 3 | 2 | 1 | 1 | 3 | | 2 | 2 | 0 | 2 | 2 |
| Diarrhoea | 2 | 2 | | 1 | 1 | 1 | 1 | | | 0 | 0 | 0 |
| Myalgia | 1 | 2 | | 0 | 1 | 2 | 4 | 1 | | 1 | 2 | 2 |
| Dyspnoea | | 1 | 1 | 0 | 0 | 2 | | | 1 | 0 | 0 | 1 |
| Arthralgia | | 1 | 1 | 1 | 0 | 1 | 2 | 2 | | 0 | 0 | 4 |
| Paraesthesia | 1 | | | 1 | 0 | 0 | | | | | | |

I, Number of patients overall with improvement in symptoms on interferon; NC, number of patients overall with no change in symptoms on interferon; W, number of patients overall with worsening of symptoms on interferon.

© Cancer Research Campaign 1998

Table 3 Immunopharmacology. Median values (and ranges) for Linomide 5 mg cohort

| Weeks from baseline | n | WBC | Neutrophils | Lymphocytes | Monocytes | Platelets | CD4+ | CD4+DR+ | CD14+ | CD14+DQ+ | CD56+CD3− |
|---|---|---|---|---|---|---|---|---|---|---|---|
| 0 | 14 | 8.9 (6–11.1) | 6.7 (3.7–8.7) | 1.1 (0.3–2.5) | 0.65 (0.36–1.3) | 380 (211–826) | 492.5 (165–2541) | 74.5 (18–518) | 674 (284–1774) | 35 (9–655) | 95.5 (10–311) |
| 2 | 13 | 11.6* (7.6–25.1) | 8.75* (5.6–23.6) | 1.45 (0.5–2.4) | 0.7* (0.3–1.8) | 446* (226–960) | 410 (124–1837) | 60* (14–226) | 665 (429–1563) | 118 (0–365) | 54 (7–127) |
| 8 | 7 | 6.35 (4.5–9.2) | 4.8 (3.6–6.5) | 0.73 (0.5–1.9) | 0.38 (0.2–1) | 259.5* (100–353) | 670 (85–1135) | 52 (8–137) | 709 (187–818) | 137 (6–218) | 41 (8–172) |

*Significantly different from baseline by Wilcoxon signed rank sum test ($P < 0.05$).

myalgia and somnolence was seen with Linomide treatment alone, again worsening with the addition of α-interferon. Of the 27 patients who received α-interferon, four experienced thrombocytopenia during treatment with α-interferon and Linomide (one grade 1, three grade 2). During treatment, there was a tendency to a fall in haemoglobin levels, with 17 patients developing some degree of anaemia (eight grade 1, eight grade 2 and one grade 3).

There were no treatment-related deaths. One patient died on study from progressive disease. Nine patients experienced serious adverse events possibly related to Linomide, five of whom discontinued Linomide: two with confirmed, exudative pericarditis, one with a pleural effusion combined with gastrointestinal disturbances, one with Guillian–Barré syndrome and the fifth with lethargy and leg pain. Of the remaining four patients who continued Linomide, two had anaemia, one gastrointestinal disturbance and one suspected, non-exudative pericarditis.

### Treatment duration and doses of α-interferon

Median treatment duration was 8 weeks, including 6 weeks of α-interferon with Linomide (range 1–81 weeks). Treatment was discontinued in 13 patients because of disease progression after a median of 8 weeks (range 2–19), and in ten patients because of toxicity after a median of 7 weeks (range 1–12). Of the remaining eight patients, four stopped therapy because of 'completion of study' (after a median of 33 weeks, range 16–81), and four at patient's or clinician's request with toxicity as a contributing event after a median of 9 weeks treatment (range 2–12).

The first cohort of 17 patients received 5 mg of Linomide. Of these, three did not start α-interferon, two because of toxicity and one of progressive disease. After the first eight patients, the maximal dose of α-interferon was reduced from 12 to 9 MU because 12 MU was not tolerated. Overall, with 5 mg of Linomide, 11 patients reached a dose of at least 9 MU of α-interferon three times a week and were treated for a median of 11 weeks.

The second cohort of 14 patients received 10 mg of Linomide, one of whom did not start α-interferon because of disease progression. Of the 13 patients receiving α-interferon, 12 received the maximal planned dose of 9 MU of α-interferon with a median treatment duration of 10 weeks. Five patients subsequently required dose reductions of α-interferon and three required dose reductions of Linomide owing to side effects.

### Objective responses

Response was evaluable in 22 patients (13 on 5 mg and nine on 10 mg of Linomide). There was one complete remission after 36 weeks treatment in a 57-year-old male with lung metastases from renal cell carcinoma in the 10-mg group. He continued treatment for 56 weeks and stopped because of completion of study, but progressed 17 weeks later. Six patients (three renal and three melanoma) had stable disease, with a median time to progression of 18 weeks (range 12–81 weeks). Overall median time to progression was 9 weeks (range 2–81 weeks) for the 5-mg group and 12 weeks (3–73 weeks) for the 10-mg group.

### Immunopharmacology

Nineteen patients had immunopharmacology blood samples taken at baseline (14 in the 5-mg group, five in the 10-mg group). Statistical tests were performed on 13 patients between baseline and 2 weeks and on seven patients between baseline and 8 weeks, with follow-up blood samples in the 5-mg group only. No such tests were performed on the 10-mg group owing to limited numbers ($n = 5$). Results are shown in Table 3 for the 5-mg Linomide group. There was a statistically significant decrease in the absolute number of activated T-helper cells ($CD4^+DR^+$) after 2 weeks of 5 mg Linomide, but these changes were not sustained after 8 weeks of combined treatment. No change in the absolute and relative number of monocytes ($CD14^+$), activated monocytes ($CD14^+DQ^+$), or NK cells ($CD56^+CD3^-$) was noted with treatment. In 12 patients tested, there was no effect on the NK- and lymphokine-activated killer (LAK) cell activity during treatment.

There was a significant increase in the number of WBCs and neutrophils during the first 2 weeks of treatment with Linomide in both dose groups and in monocytes in the 5-mg Linomide group, but this was not sustained to 8 weeks when α-interferon was added. The number of lymphocytes showed a significant decrease after 8 weeks in the 10-mg Linomide group alone. The number of platelets increased significantly after 2 weeks, but then decreased significantly after 8 weeks of treatment in both dose groups.

### DISCUSSION

This is the first time α-interferon and Linomide have been used together. We observed no change in the absolute and relative number of NK cells after Linomide alone, nor when α-interferon was added. This was in contrast to previous preclinical and clinical studies (Kalland et al, 1985*a*; Kalland, 1990; Bengtsson et al, 1992; Bergh et al, 1997), which have shown an increase in absolute and relative numbers of NK cells with an increase in functional activity during Linomide treatment. We saw a statistically significant decrease in the number of activated T-cells ($CD4^+DR^+$) after 2 weeks of treatment with Linomide alone, not sustained after 8 weeks of combined treatment. In two previous clinical studies with Linomide (Bengtsson et al, 1992; Bergh et al,

© Cancer Research Campaign 1998

1997), no effect had been seen on activated T-cells. There was no change in monocytes during treatment in this study compared with increases in absolute and relative numbers of monocytes previously reported (Bengtsson et al. 1992; Bergh et al. 1997).

Preclinical studies have demonstrated that enhanced NK cell activity during Linomide treatment is due to recruitment of new cells from precursor cells in the bone marrow, rather than an increase in the cytotoxic activity of pre-existing cells (Kalland, 1990). We saw no change in LAK and NK cell cytotoxicity. The apparent lack of effect on immunological parameters in this study, particularly NK cell numbers and activity, may be a result of the gross impairment of the immunological system in patients with advanced cancer. Indeed, patients with metastatic disease often have abnormalities in NK cell function and/or NK cell numbers (Whiteside et al. 1994).

The frequency of adverse events during the first 2 weeks of treatment with Linomide alone appears to be the same in both dose groups. When α-interferon was added, the frequency and severity of toxicity in each group increased. Combined treatment was poorly tolerated, with 45% of the patients stopping therapy because of toxicity as a primary (ten patients) or contributing reason (four clinician/patient request). This is much higher than the 6% withdrawal rate for toxicity reported with α-interferon alone at doses greater than 9 MU three times weekly (Minasian et al. 1993). The similar adverse event profile of Linomide and α-interferon (fever, fatigue, myalgia, flu-like symptoms, nausea and vomiting) probably explains the low tolerance of the combination in these patients.

In an earlier study, single-agent Linomide 15 mg given twice weekly achieved two minor responses in renal cell carcinoma (Bergh et al. 1997). The disappointing observation of only one response in 22 evaluable patients (4.5%) in the current study makes it unlikely that the true response rate is over 20% ($P = 0.048$, one-sided test). The lack of added efficacy with the combination may be due to the absence of the anticipated immunological benefits, particularly an increase in number and functional activity of NK cells. This observation, together with the poor tolerability, leads us to conclude that the combination of α-interferon and Linomide does not merit further exploration using these doses and this schedule.

### ACKNOWLEDGEMENT

This study was supported by Pharmacia and Upjohn AB, Clinical Trial Number T1223118.

### REFERENCES

Armitage P and Berg G (1987) *Statistical Methods in Medical Research*, 2nd edn. Blackwell: Oxford, pp. 410–411 and 428–438.

Bengtsson M, Simonssom B, Carlsson K, Nilsson B, Smedmyr B, Termander B, Oberg G and Totterham TH (1992) Stimulation of NK cell, T cell and monocyte functions by the novel immunomodulator linomide after autologous bone marrow transplantation. A pilot study in patients with acute myeloid leukaemia. *Transplantation* **53**: 882–888

Bergh JCS, Totterman TH, Termander BC, Standgurden KA, Gunnarsson PO and Nilsson BI (1997) The first clinical pilot study of roquinimex (Linomide) in cancer patients with special focus on immunological effects. *Cancer Invest* **15**: 204–211

Harning R and Szalay J (1988) A treatment for metastasis of murine ocular melanoma. *Invest Ophthal Visual Science* **29**: 1505–1510

Harning R, Koo GC and Szalay J (1989) Regulation of the metastasis of murine ocular melanoma by natural killer cells. *Invest Ophthal Vis Science* **30**: 1909–1915

Horoszewicz JS and Murphy GP (1989) An assessment of the current use of human interferons in therapy of urological cancers. *J Urol* **142**: 1173–1180

Kalland T (1986) Effects of the immunomodulator LS 2616 on growth and metastasis of the murine B16-F10 melanoma. *Cancer Res* **46**: 3018–3022

Kalland T (1990) Regulation of natural killer progenitors. Studies with a novel immunomodulator with distinct defects at the precursor level. *J Immunol* **144**: 4472–4476

Kalland T, Alm G and Stalhandske T (1985*a*) Augmentation of mouse natural killer cell activity by LS 2616, a new immunomodulator. *J Immunol* **134**: 3956–3961

Kalland T, Makismova A and Stalhandske T (1985*b*) Prophylaxis and treatment of experimental tumours with the immunomodulator LS 2616. *Int J Immunopharmacol* **7**: 390

Kirkwood JM (1991) Studies of interferons in the therapy of melanoma. *Semin Oncol* **18** (5)(suppl. 7): 83–90

Larsson EL, Joki A and Stalhandske T (1987) Mechanism of action of the new immunomodulator LS 2616 on T cell responses. *Int J Immunopharm* **9**: 425–431

Lesko MJ, Lever RS, Mackie RM and Parrott DMV (1989) The effect of topical steroid application on natural killer cell activity. *Clin Exper Allergy* **19**: 633–636

Miller AB, Hoogstraten B, Staquet M and Winkler A (1981) Reporting results of cancer treatment. *Cancer* **47**: 207–214

Minasian LM, Motzer RJ, Gluck L, Mazumdar M, Vlamis V and Krown SE (1993) Interferon alpha-2a in advanced renal cell carcinoma: treatment results and survival in 159 patients with long-term follow up. *J Clin Oncol* **11**: 1368–1375

Oliver RTD (1994) Is there long term survival advantage from cytokine treatment? *Eur J Cancer* **30A**: 1214–1216

Stalhandske T and Kalland T (1986) Effects of the novel immunomodulator LS 2616 on the delayed-type hypersensitivity reaction to Bordetella pertussis in the rat. *Immunopharmacology* **11**: 87–92

Stalhandske T, Erikson E and Sandberg BM (1982) A novel quinolone-carboxamide with interesting immunomodulatory activity. *Int J Immunopharmacol* **4**: 336

Whiteside TL and Heberman RB (1994) Role of human natural killer cells in health and disease. *Clin Diag Lab Immunol* **1**: 125–133

© Cancer Research Campaign 1998